# A patient-centered, theory-guided approach to examining the barriers and enablers to trial participation among people with SCD

**DOI:** 10.1093/jscdis/yoaf032

**Published:** 2025-10-07

**Authors:** Kelly Carroll, Natasha Hudek, Justin Presseau, Lanre Tunji-Ajayi, Dawn P Richards, Susan Marlin, Jamie C Brehaut

**Affiliations:** Methodological and Implementation Research, Ottawa Hospital Research Institute (OHRI), The Ottawa Hospital, General Campus, Ottawa, ON K1H 8L6, Canada; Methodological and Implementation Research, Ottawa Hospital Research Institute (OHRI), The Ottawa Hospital, General Campus, Ottawa, ON K1H 8L6, Canada; Methodological and Implementation Research, Ottawa Hospital Research Institute (OHRI), The Ottawa Hospital, General Campus, Ottawa, ON K1H 8L6, Canada; School of Epidemiology and Public Health, University of Ottawa, Ottawa, ON K1G 5Z3, Canada; Sickle Cell Awareness Group of Ontario (SCAGO), North York, ON M9L 2Y8, Canada; Clinical Trials Ontario, MaRS Centre, Toronto, ON M5G 1M1, Canada; Clinical Trials Ontario, MaRS Centre, Toronto, ON M5G 1M1, Canada; Methodological and Implementation Research, Ottawa Hospital Research Institute (OHRI), The Ottawa Hospital, General Campus, Ottawa, ON K1H 8L6, Canada; School of Epidemiology and Public Health, University of Ottawa, Ottawa, ON K1G 5Z3, Canada

**Keywords:** SCD, recruitment, mixed methods, theoretical domains framework, theory, trial participation

## Abstract

**Objective:**

Recruitment to clinical trials involving sickle cell disease (SCD) patients can be challenging, leaving trialists uncertain about how to optimize recruitment approaches and strategies. Informed by the Theoretical Domains Framework (TDF), we identified a comprehensive set of barriers and enablers to participation in SCD trials, and suggest how this theory-informed survey approach can improve trial recruitment strategies.

**Methods:**

In collaboration with Clinical Trials Ontario and Sickle Cell Awareness Group of Ontario (SCAGO), we conducted a mixed methods study involving interviews with and surveys of SCD patients and families. We iteratively adapted a template survey based on think-aloud interviews, before administering the adapted survey online to SCAGO membership.

**Results:**

Fifteen interviews with SCAGO members led to 49 survey items across 13 of 14 TDF domains. Four new items specific to the SCD community were added. Administration challenges led to low survey response, with only 22 people completing the survey. Eighteen items from 8 domains were seen as barriers (eg invasive tests/procedures, travel to study site). Twenty-two items from 9 domains were seen as enablers (eg hope for a cure, helping others).

**Conclusion:**

Our theory-guided approach identified a comprehensive set of factors related to SCD trial participation, information that can support recruitment strategy development prior to trial onset. Low survey response rates precluded strong conclusions about the relative priority of the individual barriers and enablers; more work will be needed among a broader sample of SCD patients and families. Identification of theory-guided behavioral domains offers targeted suggestions for trial recruitment.

## INTRODUCTION

Sickle cell disease (SCD) is an inherited red blood cell disorder caused by abnormal Hb, primarily affecting people of African ancestry.^1^^,^^2^ It is often associated with pain, infections, ACS, and stroke,^3^ frequently resulting in hospital admissions and emergency department visits.^4^ An estimated 70 000 to 100 000 Americans have SCD,^5^ affecting about 0.27% of all Black or African-American births.^3^ Based on a 2023 census, there are 3418 patients with SCD in Ontario, Canada,^6^ and approximately 6000 people across Canada living with SCD.^7^

Potentially curative treatments for SCD include bone marrow transplantation, which require available donors, and very new gene therapies, which can come with significant risks and are not yet widely available.^8^ As such, SCD has been essentially incurable for the majority of those affected.^9^ Treatment for SCD typically focuses on management of acute symptoms and/or delaying or preventing chronic organ damage.^10^ New treatments for SCD have been slow to be developed despite considerable effort, and vary widely in effectiveness.^11^ When promising innovations do emerge, encouraging participation in clinical trials can be a challenge. Slow recruitment, or even termination of trials due to poor recruitment, is common.^10^^,^^11^ There are many known factors impeding recruitment to SCD trials, including limited access to SCD-specific trial information,^2^ uncertainty about long-term risks and side effects,^12^ concerns about enrolling in randomized trials,^10^ and unmanageable trial demands.^12^ Furthermore, there has been longstanding distrust stemming from past unethical research practices and structural racism in health care.^10^^,^^13^ The latter has contributed to many members of the SCD community being distrustful of research and the healthcare system in general^11^^,^^14^; and mistrust among the SCD community has been cited to be a particularly important barrier to clinical trial participation.^2^^,^^10–12^^,^^14^

Slow or inadequate trial participation can have significant long-term consequences for health research, including wasted resources, opportunity costs, limited generalizability of findings, ethical concerns related to exposing participants to risk without scientific gain, and potential erosion of public trust.^15^ However, the potential for delayed access to much-needed new treatments is particularly salient in SCD. Identifying barriers to and enablers of participation *prior to trial onset* can enable trialists to account for these factors, and design tailored recruitment strategies that can increase trial recruitment and improve participant experience.^16^

We have developed a patient informed, condition-specific, and theory-guided approach to surveying potential participant populations about factors relevant to trial participation. We have previously described the development of this approach,^16^ applied it to breast cancer^17^ and Huntington’s disease^18^ patient populations, and developed user-friendly guidance for its implementation.^19^ Here, we apply this method to the context of participation in SCD clinical trials.

Our approach to designing an adaptable, survey-based method for identifying barriers to/enablers of clinical trial participation in SCD was informed by the Theoretical Domains Framework (TDF).^20–22^ The TDF organizes over 100 constructs/factors known to be related to behavior and behavior change into 14 domains that capture determinants of health behaviors. Understanding these domains can suggest change strategies, since domain-specific change strategies are known for other types of behavior.^16^ Our previous work demonstrated that this approach uncovers a broader range of barriers and enablers compared to existing survey-based methods, identifies relevant factors from a wider range of behavioral domains than other survey-based approaches,^16^ and most importantly, can provide specific guidance for tailored recruitment strategies that account for these factors.

In this paper, we aimed to assess knowledge of clinical trials, identify barriers to and enablers of trial participation in SCD-specific research, and illustrate how a theory informed survey can support the design of more targeted recruitment strategies.

## MATERIALS AND METHODS

This work was reviewed and approved by the Ottawa Health Science Network Research Ethics Board (Protocol # 20180250). We followed the CHERRIES guidance specifically for the survey component for reporting online surveys.^23^

This work was conducted in collaboration with Clinical Trials Ontario (CTO), a not-for-profit organization dedicated to improving the clinical trial environment in Ontario, Canada. CTO is working with various patient organizations, health charities, and health partners on various initiatives, including the design and administration of surveys for patients and community members. The goal of these surveys is to assess members’ knowledge of, attitudes toward, and participation in clinical trials and clinical research. For this work, we also collaborated with Sickle Cell Awareness Group of Ontario (SCAGO) to develop a theory informed online survey assessing the barriers to and enablers of trial participation.

### Previous survey template development

Our approach to developing these surveys has been fully described elsewhere.^16^ In summary, we used the Theoretical Domains Framework (TDF) to guide the development of a template survey exploring barriers to and enablers of clinical trial participation. Template items were first developed through a literature search for barriers and enablers relevant to each of the 14 TDF domains: Beliefs About Consequences, Social Influences, Reinforcement, Goals, Beliefs About Capabilities, Environmental Context and Resources, Skills, Social/Professional Roles and Identity, Knowledge, Optimism, Memory, Attention and Decision Processes, Emotions, Behavioral Regulation. Interviews with patient and family members of 2 other health conditions were sequentially conducted to ensure clarity of the survey template, to obtain opinions on the included barriers and enablers, and to identify additional condition-specific factors.^17^^,^^18^

### SCD-specific interviews

For this study, we began with items from this template as our draft version (Table S1). We then conducted interviews to tailor the survey specifically to identify barriers and enablers relevant to trial participation in the SCD community. Wording modifications were made to reflect the current clinical condition and setting, and study team members with expertise in SCD reviewed the survey and provided feedback.

SCAGO then approached membership patients and family members with lived experience with SCD, inviting them to participate and obtaining permission to contact. All individual interviews were conducted via Zoom video conferencing with 2 study team members (K.C. and N.H.). Think-aloud interviews were conducted, in which interviewees were asked to have the draft survey in front of them (sent via email in advance) and to read aloud as they worked through the survey, verbalizing their thoughts and impressions as they read instructions, interpreted questions, selected responses, and provided feedback. Both interviewers took notes, which served as the basis for discussions between study team members between sessions. All sessions were audio recorded. Changes were made iteratively to the interview document every 2-3 interviews.

### Survey content

After the pilot interviews were completed and all changes to the paper document were made, an anonymized web-based version of the survey was created and tested by the study team to ensure functionality, that instructions were clear, and that data were being captured correctly. The anonymous online survey was programmed by the Ottawa Hospital Methods Centre using Microsoft Visual Basic and a SQL Server database. It was hosted on a secure server within The Ottawa Hospital’s data center. The survey and support materials—including participant information sheets and mailouts were translated and administered in both English and French. The survey included 3 sections. Section 1 collected information about respondents’ SCD status, research experience, and demographics. Disease status questions included whether they had been diagnosed with SCD themselves, whether anyone in their family or close to them had SCD (and what their relationship was to them), when they were diagnosed, and questions about SCD-related pain. If respondents indicated that they did not have SCD, but their child or someone else close to them did, question wordings were adapted accordingly. Research experience questions included whether they had ever been approached to take part in SCD research, whether they had actually participated, and whether they—or on behalf of their child or loved one—had ever actively searched for a clinical trial in which to participate. Demographic questions collected information on postal code, age, gender, education, household income, work status or main activity, ethnic background, and the language first learned at home and still understood.

Section 2 of the survey focused on knowledge about clinical trials and asked respondents if they could confidently explain what a clinical trial was to a friend or family member. This section included 14 items adapted from the objective knowledge component of the Quality of Informed Consent (QuIC) instrument.^24^ Respondents indicated if they agreed, disagreed, or were unsure about each statement. A summary score was calculated by taking the sum for each correct/incorrect statement identified.

Section 3 of the survey included 49 potential barriers to and drivers of trial participation. Respondents were asked to rate how each factor might influence their decision—specifically, whether it would push them AWAY from participating (*a little or a lot*), push them TOWARDS participating (*a little or a lot*), or have no effect. The survey did not allow backtracking, and responses were saved immediately upon clicking the “Next” button. Unique site visitors were tracked using a session ID generated as soon as respondents clicked the “Start the survey” link at the bottom of the welcome section.

### Survey administration

SCAGO advertised the survey in both English and French via its social media platforms (Facebook, Instagram, WhatsApp, its website), its biweekly Newsletters, and in an invitation email to members of its subscriber list. As such, a prenotification advertisement was first posted in their September 30, 2022 Newsletter emailed to members on its subscriber list. On October 6, 2022, members received an invitation email containing a brief description of the survey along with a link to the online questionnaire. A link to the REB approved participant information sheet was included on the landing page of the survey. At this time, the study was also advertised on its social media platforms. A follow-up email was sent 2 weeks later. The study continued to be advertised in their biweekly Newsletter and on social media until the study was taken offline on March 29, 2023. SCAGO also shared the survey link with their other Canadian collaborators. No monetary incentives were provided.

Data were downloaded to Microsoft Excel for cleaning and imported into SPSS for analysis. Data from participants with the same IP address were retained if their responses were not identical, allowing multiple individuals with SCD and caregivers from the same household to participate. Duplicate entries in the database were identified by examining session IDs and timestamps, with the most recent or most complete record retained for analysis.

#### Analysis

*Pre-survey interviews:* Frequencies summarized demographic, disease, and trial experience variables for the interviewees. Clarifications, modifications, and the addition of new items suggested by interviewees were incorporated into subsequent iterations of the interviews and then into the final version of the survey.

*Survey*: For the online survey data, descriptive analyses included frequencies and percentages for all demographic, experience, and knowledge variables, as well as for ratings of barriers and enablers to trial participation. For these ratings, items were classified as “barriers” or “enablers” or “having no effect” according to the highest percentage of respondents endorsing that response. Items endorsed as a barrier by at least 25% of respondents and as an enabler by at least 25% (an arbitrary cutoff) were categorized as having a “varied response.” Means and standard deviations were calculated for continuous variables. All analyses were conducted using SPSS (version 29).^25^

## RESULTS

### Pre-survey interviews

Table 1 describes interviewees’ demographic details and trial experience. Some data are missing for these items because the interviews were focused on obtaining opinions about the upcoming survey, eg think-aloud participants who read a demographic question and clearly understood it but failed to answer it were not pressed to answer. Furthermore, some demographic items were modified/added/removed between interviews. Fifteen pilot interviews were conducted with members of SCAGO, with the majority self-identifying as women (12; 80%), ranging in age from 22 to 72 years old (Mean = 39). Most (n = 14; 93%) indicated a college or undergraduate degree or higher. Most interviewees were Black African (n = 7; 47%) or Black Caribbean (n = 5; 33%). Almost half (n = 7; 47%) reported being diagnosed with SCD themselves, and most (n = 13; 87%) indicated having family members with SCD. Of these, 8 (62%%) had a child with SCD and 4 (31%) had a sibling with the disease.

**Table 1.** Pre-survey interviews: self-reported demographics and SCD-specific characteristics (N = 15).

Demographics	N (% Total)
**Gender**	
Men	3 (20.0)
Women	12 (80.0)
Transgender	0 (0)
Prefer to self-identify	0 (0)
**Age**	Range (22-72) M (SD) = 39.5 (13.4)
**Education**	
High school or less	1 (6.7)
College diploma/BA degree	10 (66.7)
Post graduate or professional degree	4 (26.7)
**Ethnicity** ^a^	
Black Caribbean	5 (33.3)
Black African	7 (46.7)
South Asian	1 (6.7)
Arab	1 (6.7)
Missing	1 (6.7)
**Respondent diagnosed with SCD**	
Yes	7 (46.7)
No	8 (53.3)
**Family members/or someone close have been diagnosed with SCD**	
Yes	13 (86.7)
No	2 (13.3)

a Multiple selections possible.

Table 2 describes reported experience with and knowledge about clinical trials among our interview respondents. Eight (53%) indicated that they or their loved one had been approached to participate in a research study about SCD, and 8 (53%) indicating participation in a research study. The most common type of research participation included an interview (n = 5; 62%), database study (n = 5; 62%), or survey (n = 4; 50%), with 2 (25%) being a clinical trial. Only 3 (20%) looked for a clinical trial to participate in. Confidence about whether they could explain what a clinical trial was to a friend or family member was high, with 87% expressing confidence and 13% reporting that they were not confident.

**Table 2.** Reported experience with and knowledge about clinical trials among interviewees (N = 15).

Question	N (% Total)
Responded “yes” to being approached for research	8 (53.3)
Responded “yes” to ever participating in research	8 (53.3)
** What did participation involve?** ^a^	
Clinical trial	2 (25.0)
Survey	4 (50.0)
Interview	5 (62.0)
Database study	5 (62.0)
Other	1 (6.7)
**Responded “yes” to having actively looked for a clinical trial**	3 (20.0)
**Confidence in clinical trial knowledge?**	
Not at all confident	1 (6.7)
Not very confident	1 (6.7)
Somewhat confident	10 (66.7)
Completely confident	3 (20.0)

a Multiple selections could apply.

Table S1 summarizes specific barrier/enabler item changes from initial to final iteration during the interview process. For the barrier/enabler items, 47 items were included from our template,^18^ which had previously targeted a chronic, rare disease (Huntington’s disease). Between interview data and final survey administration, 6 items were modified for clarity. Two items from the “Beliefs About Consequences” domain were modified to be more specific to SCD (eg *If I had to have more tests; if I had to have more invasive tests/procedures [eg bone marrow transplant]*). One item was deleted because it was specific to a different condition. Four items were added based on multiple interviewee suggestions (“Social Influences” domain: *if sickle cell patient organizations support trial participation, if people I follow on social media support trial participation*, “Beliefs about Consequences” domain: *if the study documents mention a risk of death during the study*, “Social Influences” domain: *if there was access to study staff that speaks my native language*). Two additional items were identified in the interviews, but could not be implemented in a non-setting-specific survey (“Social Influences” domain: *my level of trust in the research team, if the study staff shares my cultural background*). One item was adapted for this setting by combining multiple items from a previous survey (“Social Influences” domain: *my worry that participation would mean that others would find out about my condition [eg friends, family, co-workers]*).

### Survey data

**Figure 1.**
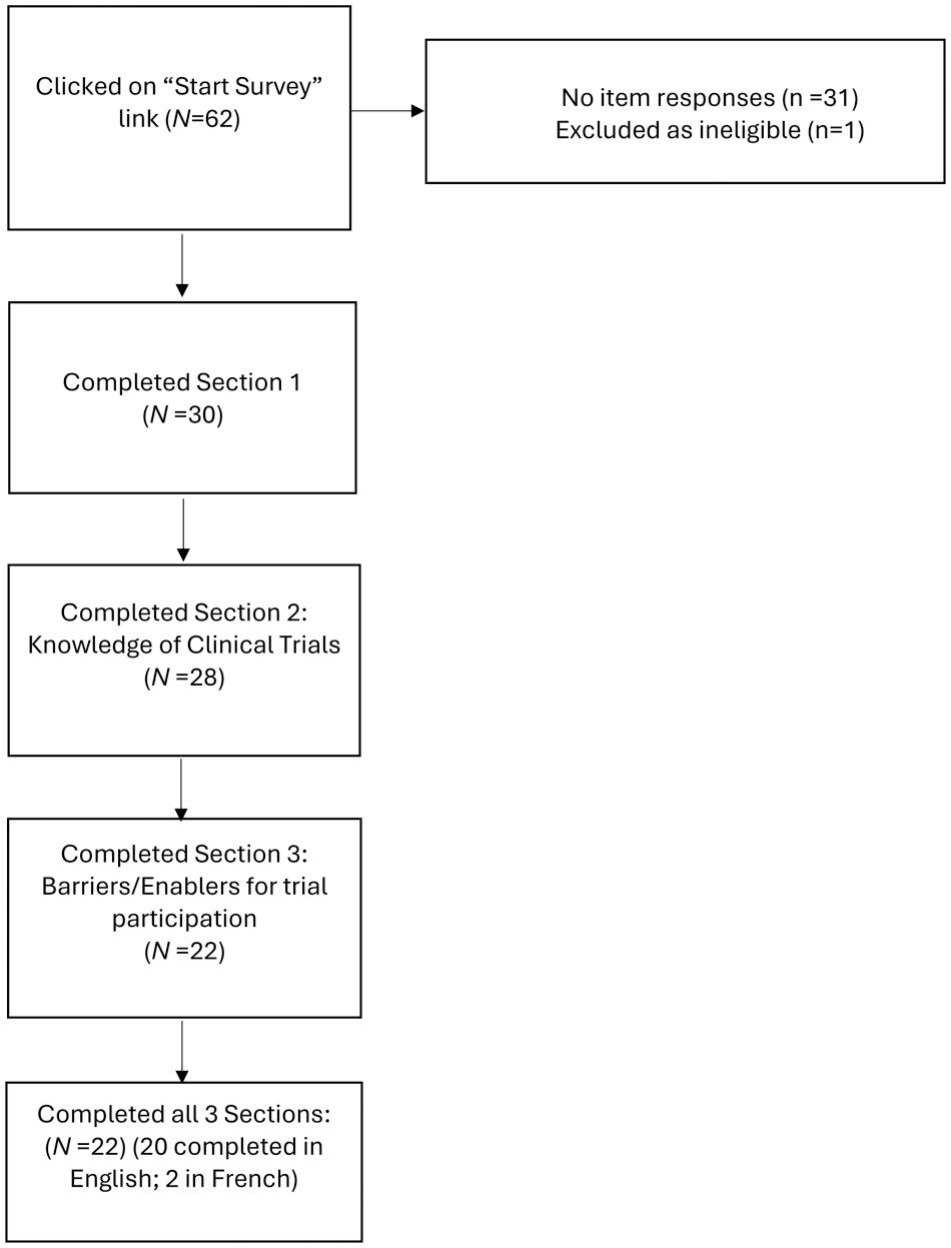
Survey study flow diagram.

Figure 1 describes the response flow diagram for our survey. Among the SCAGO subscribers that received notices about the survey, 62 clicked “Start Survey,” 30 completed the initial demographics section of the survey, and a total of 22 completed all 3 sections of the survey. The majority completed the survey in English (n = 20; 91%).

Table 3 summarizes the self-reported demographic and SCD-specific characteristics of our survey respondents. Most identified as women (n = 21; 70%), with a mean age of 41 years (range 21-74). The majority were living in Ontario (n = 20; 67%). Most (n = 25; 83%) indicated a college or undergraduate degree or higher, and 14 (47%) reported household incomes of $50 000 or more. Most respondents were Black Caribbean (n = 15; 50%) or Black African (n = 8; 27%), spoke English at home (n = 23; 82%), and were working full-time (13; 43%). Most reported having SCD themselves (n = 21; 70%), and 18 (60%) reported having family members or someone else close to them with SCD. Of these, 6 (33%) reported having a child with SCD and 8 (44%) reported having another family member with the disease. Thirty percent (n = 9) of respondents experienced SCD-related pain monthly over the course of the last year, while another 10 (33%) reported pain less than 5 times in the year. Thirty-three percent (n = 10) reported that SCD-related pain kept them from work, school, household tasks, or from seeing family and friends monthly within the last year, while 11 (37%) reported this happening less than 5 times within the year. Fifty-seven percent (n = 17) indicated that they had at least 1 hospital visit in the last year due to SCD-related pain. Most respondents reported being diagnosed with SCD after birth (n = 16; 56%), or during routine newborn screening (n = 11; 37%). Most respondents indicated hearing about the survey from SCAGO (n = 27; 90%).

**Table 3.** Survey: self-reported demographics and SCD-specific characteristics (N = 30).

Demographics	N (% Total)
**Gender**	
Men	7 (26.7)
Women	21 (70.0)
Transgender	0 (0)
Non-binary	1 (3.3)
Missing	1 (3.3)
**Age**	Range (21-74) M (SD)=41.3 (13.1)
**Geographic location (from postal codes provided)**	
Ontario	20 (66.7)
Quebec	2 (6.7)
British Columbia	1 (3.3)
Missing/can’t be determined	7 (23.0)
**Education**	
High school diploma only or some college/university	4 (13.3)
College diploma/BA degree	14 (46.7)
Post graduate or professional degree	11 (36.6)
Missing	1 (3.3)
**Household income**	
Less than $50 000	11 (36.7)
More than $50 000	14 (46.7)
Prefer not to answer	5 (16.7)
**Ethnicity** ^a^	
Black Caribbean	15 (50.0)
Black African	8 (26.7)
Black Canadian	3 (10.0)
Other	5 (16.7)
**Language first learned at home in childhood and can still understand?** ^a^	
English	23 (82.1)
Yoruba	2 (7.1)
Other	3 (10.8)
Missing	2 (6.7)
**Employment** ^a^	
Full-time employment	13 (43.3)
Self employed	7 (23.3)
Part-time employment	5 (16.7)
Long-term disability	4 (13.3)
Retired	2 (6.7)
Other (eg volunteering, school, caregiving, maternity/paternity leave)	11 (36.6)
Prefer not to answer	3 (10.0)
**Respondent diagnosed with SCD**	
Yes	21 (70.0)
No	7 (23.0)
Missing	2 (6.7)
**Family members/or someone close have been diagnosed with SCD**	
Yes	18 (60.0)
No	8 (26.7)
Missing	4 (13.3)
*If Yes, Relationship to respondent*	
Respondent’s child	6 (33.3)
Other family member	8 (44.4)
Other	2 (11.1)
Missing	2 (11.1)
*Age of child with sickle cell*	Range (4-15 yrs.)
**SCD related pain in last year**	
Daily	4 (13.3)
Weekly	3 (10.0)
Monthly	9 (30.0)
< 5 times/year	10 (33.3)
Other	1 (3.3)
Missing	3 (10.0)
**SCD related pain kept respondent from work, school, housework, family/friends**	
Daily	1 (3.3)
Weekly	2 (6.7)
Monthly	10 (33.3)
< 5 times/year	11 (36.7)
Other	3 (10.)
Missing	3 (10.0)
**SCD related pain led respondent to hospital in last year**	
No visits	9 (30.0)
1 time	7 (23.3)
2-4 times	7 (23.3)
5 or more	3 (10.0)
Don’t know	1 (3.3)
Missing	3 (10.0)
**When diagnosed with SCD**	
Before birth	0 (0%)
At birth during routine newborn screening	11 (36.7)
After birth	16 (56.3)
Missing	3 (10.0)
**Where did respondent hear about the study?**	
SCAGO	27 (90.0)
Quebec Sickle Cell Disease Association	1 (3.3)
Sickle Cell Awareness Network of Saskatchewan	1 (3.3)
Other	1 (3.3)

a Multiple selections possible.

Table 4 describes self-reported experience with and knowledge about clinical trials. Nearly half (n = 13; 43%) of respondents indicated being approached to participate in any research study about SCD, 37% (n = 11) had participated, most commonly in previous surveys (n = 7; 64%). None reported clinical trial participation, and only 23% (n = 7) reported having actively looked for a clinical trial to participate in. Most self-reported at least some confidence in clinical trial knowledge (n = 25; 83%).

**Table 4.** Reported experience with and knowledge about clinical trials among survey respondents (N = 30)

Question	**N (% Total**)
Responded “yes” to being approached for research	13 (43.3)
Responded “yes” to ever participating in research	11 (36.7)
** What did participation involve?** ^a^	
Clinical trial	0 (0)
Survey	7 (63.6)
Interview	4 (36.4)
Database study	4 (36.4)
Don’t know	0 (0)
Other	1 (9.1)
**Responded “yes” to having actively looked for a clinical trial**	7 (23.3)
**Confidence in clinical trial knowledge?**	
Not at all confident	3 (10.0)
Not very confident	1 (3.3)
Somewhat confident	16 (53.3)
Completely confident	9 (30.0)
Missing	1 (3.3)

a Multiple selections possible.

Performance on our items assessing knowledge about clinical trials showed a mean correct score of 9 out of 14 (64%) across 28 participants who completed that section, with scores ranging from 0 to12.

#### Barriers and enablers to trial participation

Of the 49 items identified through the development process (see Table 5), 10 represented the domain of **Beliefs about Consequences,** which aims to identify issues respondents perceive as potential outcomes of participation. Among these, 4 items were seen as enablers of participation (helping others, contributing to science, helping with my condition, receiving better care), 5 were mostly perceived as barriers (longer hospital stays, potential impact on insurance coverage, increased burden on family, more invasive tests or procedures, mention of risk of death), and 1 item was viewed as having no effect (more tests). Eleven of the 49 items fell under the domain of **Social Influences,** which explores how other people may impact the decision to participate. Six of these items were identified as enablers of participation (investigators providing regular study updates, having helpful people to assist with participation decisions, family support for participation, physician encouragement, support persons to help throughout the trial, and endorsement from sickle cell patient organizations). Two items were cited as barriers: physicians receiving financial support to recruit patients and feelings about whether trial funders can be trusted. Three items were reported as having no effect: access to study staff who speak my native language, concerns about others finding out about one’s condition, and support for trial participation from people followed on social media. Five items focused on methods of **Reinforcement** to reward participation, which were primarily perceived as enablers, including receiving study results, gaining access to new study drugs, reimbursement of expenses, and payment for participation. One item—experience with previous trials—was seen as having no effect. Another 5 items addressed participants’ **Goals** and how participation might affect them. All of these were most commonly rated as barriers, such as preventing other activities, interfering with other goals, impacting social life and family commitments, conflicting with childcare responsibilities, and affecting participation in other clinical trials.

**Table 5.** Survey: Barriers and enablers to participation in clinical trials (N = 22).

Statement	Perceived as a Barrier	Perceived to have No effect	Perceived as an Enabler
	**Frequency (%)**		
**Beliefs about consequences**			
My belief that participating in a trial would help others	0 (0)	2 (9.1)	**20 (90.9)**
My belief that participating would contribute to science	0 (0)	2 (9.5)	**19 (90.4)**
My hope that participation will help me with my condition	0 (0)	2 (9.5)	**19 (90.5)**
My belief that I would receive better care if I participated	0 (0)	6 (28.6)	**15 (71.4)**
If I had to stay longer in hospital	**14 (66.7)**	5 (23.8)	2 (9.5)
My worry that my insurance coverage would be affected	**12 (60.0)**	7 (35.0)	1 (5.0)
My worry that participation will cause more work for my family	**13 (65.0)**	7 (35.0)	0 (0)
If I had to have more tests	8 (38.1)	**9 (42.9)**	4 (19.1)
If I had to have more invasive tests/procedures (eg bone marrow transplant)	**14 (80.0)**	5 (25.0)	5 (25.0)
If the study documents mention a risk of death during the study	**15 (71.5)**	6 (28.6)	0 (0)
**Social influences**			
If the investigators provided regular study updates about the trial.	1 (4.5)	3 (13.6)	**18 (81.8)**
If there were helpful people on hand to help you make a participation decision	0 (0)	7 (33.3)	**14 (66.6)**
If my family thought I should participate	0 (0)	7 (31.8)	**15 (68.2)**
If my physician(s) thought I should participate	0 (0)	3 (13.6)	**19 (86.4)**
If there were study staff to provide support throughout the trial	1 (4.5)	4 (18.2)	**17 (77.3)**
If my physician was given financial support to recruit patients into the study	**11 (52.3)**	6 (28.6)	4 (19.1)
My feelings about whether the trial funders can be trusted	**11 (52.4)**	6 (28.6)	4 (19.0)
If there was access to study staff that speaks my native language	10 (47.6)	**11(52.4)**	0 (0)
My worry that participation would mean that others would find out about my condition (eg friends, family, co-workers)	5 (23.8)	**15 (71.4)**	1 (4.8)
If sickle cell patient organizations support trial participation	0 (0)	3 (14.3)	**17 (85.7)**
If people I follow on social media support trial participation	3 (14.3)	**12 (57.1)**	6 (28.5)
**Reinforcement**			
If I received the results of the study once it was complete	0 (0)	3 (14.3)	**18 (85.7)**
If I would gain access to new study drugs	1 (5.0)	4 (20.0)	**15 (75.0)**
If the study reimbursed expenses (eg parking)	0 (0)	3 (13.6)	**19 (86.4)**
If I were paid for my participation	0 (0)	5 (22.7)	**17 (77.3)**
Previous experience with other trials	2 (10.0)	**13 (65.0)**	5 (25.0)
**Goals**			
My belief that participation would prevent me from my other activities	**14 (63.6)**	8 (36.4)	0 (0)
My belief that participation would interfere with other goals of mine	**16 (76.2)**	3 (14.3)	2 (9.5)
If I think participation would affect my social life/family commitments	**13 (65.0)**	6 (30.0)	1 (5.0)
If I think participation would interfere with my childcare responsibilities	**10 (47.6)**	9 (42.9)	2 (9.6)
If I think participation would affect my activities with other clinical trials	**10 (50.0)**	8 (40.0)	2 (10.0)
**Beliefs about capabilities**			
My belief that participating would give me a sense of control over what is happening to me	1 (4.8)	**12 (57.1)**	8 (38.0)
If I think it would be a challenge getting from my home to the study site	**17 (77.3)**	3 (13.6)	3 (9.1)
If I think my overall health is good	**8 (31.8)**	**7 (31.8)**	**8 (36.3)**
My feelings about the quality of my drug plan	5 (23.8)	**12 (57.1)**	4 (19.1)
**Environmental context and resources**			
If there were patient-friendly decision-making tools to help you make a participation decision	9 (4.5)	5 (22.7)	**16 (72.8)**
If the study provided transportation to/from study appointments	0 (0)	3 (14.3)	**18 (85.7)**
If I think there is a substantial time commitment	**17 (77.3)**	3 (13.6)	2 (9.1)
My feelings about the quality of the health care system	**7 (33.3)**	**8 (38.1)**	**6 (28.6)**
**Skills**			
If I find the trial documents hard to understand	**15 (68.2)**	5 (22.7)	2 (9.1)
If the consent documents describe probabilities of side effects and numbers of patients affected by them	**14 (63.6)**	4 (18.2)	4 (18.2)
**Social/professional role & identity**			
My belief that participating would give me a sense of purpose	0 (0)	5 (23.8)	**16 (76.2)**
My belief that participating is part of being a good citizen	1 (4.5)	6 (27.3)	**15 (68.2)**
**Knowledge**			
My belief about whether I’d learn more about my condition if I participated	0 (0)	4 (18.2)	**18 (81.8)**
If the trial were testing a very new untested treatment	**11 (55.0)**	5 (25.0)	4 (20.0)
**Optimism**			
My hope that participation would help find a cure	0 (0)	1 (4.8)	**20 (95.2)**
**Memory, attention, and decision processes**			
If the investigators provided telephone reminders about study appointments	1 (4.5)	9 (40.9)	**12 (54.6)**
**Emotion**			
My worry about unknown side effects	**15 (68.2)**	3 (13.6)	3 (13.6)
**Behavioral regulation**			
My belief that trial participation would help me plan how to manage treatment better	0 (0)	5 (22.7)	**17 (77.3)**

Bold indicates largest proportion of responses.

Four of the 49 items focused on perceived **Beliefs about Capabilities**, which address whether participants feel able to participate. Among these, 1 was identified as a barrier: (getting from home to the study site), 2 were seen as having no effect (control over what is happening, quality of drug plan), and 1 elicited varied response as to whether it was a barrier or enabler (> 25% in both responses; thinking overall health is good). Four items fell under the domain **Environmental Context and Resources**, relating to aspects of the trial setting that could impact participation. Two were perceived as enablers (patient friendly decision-making tools, transportation to and from study appointments), 1 was identified as a barrier (time commitment), and 1 received varied responses as to whether it was a barrier or enabler (feelings about the quality of the healthcare system). Two barriers were related to the **Skills** needed for participation: trial documents being difficult to understand and consent forms describing probabilities of side effects and the number of patients affected by them. Two items addressed **Social/Professional Roles and Identity** and their influence on participation, both were perceived as enablers (a sense of purpose and the role as a good citizen). Two items focused on the **Knowledge** to be gained from participation or desired for decision-making: 1 was seen as an enabler (learning more about my condition), and 1 as a barrier (if the trial involved testing a very new, untested treatment). Other items included one enabler related to **Optimism** about outcomes of participation (hoping participation would help find a cure), one enabler related to **Memory**, **Attention and Decision Processes** (if investigators provided telephone reminders about study appointments), one barrier related to **Emotions** relevant to participation (worry about unknown side effects), and one enabler related to **Behavioral Regulation** (planning how to manage treatment better).

## DISCUSSION

We sought to explore the barriers to and enablers for SCD trial participation with a mixed methods interview/survey-based approach that can be administered to patient communities prior to beginning a trial. Our theory-guided approach, along with engaging patients and family members’ opinions and perspectives, identified a wide range of barriers and enablers of trial participation that could lead to specific suggestions about targeted recruitment strategies.

Our survey development process produced 49 items based on 13 theoretical domains of behavior change. Eighteen items from 8 domains were generally seen as barriers by survey respondents (eg *more invasive tests or procedures*, *travel to the study site*, *time commitment*, *interference with goals*, the *mention of risk of death in the study documents)*. Twenty-two items from 9 domains were generally seen as enablers of trial participation (eg *optimism about the likelihood of finding a cure for sickle cell disease*, *helping others*, *contributing to science).* Seven items (across 4 domains) were generally seen not to be relevant to trial participation decisions by our survey respondents (eg *needing to have more tests, study staff speaking the native language, others finding out about my condition*). Our approach was therefore effective in identifying a wide range of relevant barriers and enablers among our survey sample. Indeed, our previous work suggests it captures a fuller range of such factors compared to other frameworks in the literature.^16–18^

Note that not all items identified by interviewees were endorsed by survey respondents. Two items (*staff speaking native language* and *support from people followed on social media*) were suggested by our interviewees as potentially important enablers, but not endorsed by survey respondents. If these survey results are robust (which we cannot assume in this case due to low response rate), this would support the need to corroborate the relevance of interview-based results with larger sample surveys; issues specific to individual interviewees may not always be generalizable, perhaps because, as was the case here, some of the interviewees had actual trial experience, while the survey respondents reportedly did not.

Two items had varied responses (*if I think my overall health is good, my feelings about the quality of the health care system*), meaning that some people rated these as barriers to trial participation and others as enablers. For example, “*better health*” may make some individuals less motivated to take on the potential demands of a trial and instead prioritize other life pursuits, while others may perceive more capability to participate when their health is good. The notion that the same issue may drive to or from participation depending on individual differences is one that is rarely considered in the trial recruitment literature, and speaks to the potential need to tailor recruitment efforts to different participants individually.

One of the most commonly endorsed barriers to trial participation was the prospect of having to *have more invasive tests or procedures* (eg bone marrow transplants), while *needing to have more tests* was generally seen as not relevant. Since many SCD patients are battling chronic pain and already face many procedures, this finding may indicate a wish to avoid additional pain beyond what is already familiar to them.^10^ The importance of clinical research staff discussing these fears with patients might prove beneficial, as well as addressing any pain management that could be made available.

### TDF domains—effects on SCD trial recruitment

Consideration of the TDF theoretical domains has a number of potential benefits. First, it leverages knowledge from a large literature and wide range of theories about human behavior change to the relatively novel area of research participation. Second, it enables a more comprehensive consideration of factors relevant to participation than other recruitment-specific frameworks. Most importantly, it enables theory-guided hypotheses about how to affect participation that would not otherwise be evident.^26^^,^^27^ For example, many respondents not surprisingly identified various “Beliefs About Consequences” (eg *more invasive tests or procedures, the mention of risk of death in study documents, hope that it will help with my condition*), as important to participation. Knowing that this general domain is particularly relevant to participation decisions, we can prioritize behavior change strategies specifically effective in that domain (eg information about outcomes) and perhaps de-emphasize behavior change strategies less likely to be effective (eg goal specification, role play) to inform recruitment interventions.^26^

As another example, the “Skills” domain was also mainly identified as a barrier. Our finding that being presented with “*hard to understand trial documents*” and “*probabilities of side effects and numbers of patients affected by them*” would act as barriers suggest a skills-based issue that might benefit from providing decision support,^28^^,^^29^ education in health literacy by SCD patient organizations and health professionals, and ensuring that trial documents are easily understood and more patient friendly. During our interview sessions, it was brought to our attention that there was a desire to know about the probability information associated with a trial; however, this item also acted to push individuals away from participating. This points to a limitation of a survey-only based barriers assessment, as this makes it impossible to detect why a particular item acts as a barrier, thus requiring further discussion between researchers and patients.

While the TDF captured many factors already known to affect participation in clinical trials (eg consequences of trial participation and skills-based issues); it also served to cast a wider net on factors that may not necessarily be as obvious. For instance, respondents identified that receiving regular study updates from study investigators about the trial, ensuring support from study staff throughout the trial, and having sickle cell patient organizations endorse trial participation would all encourage participation (from “Social Influences” domain). In the “Knowledge” domain, the belief that participation would lead to greater understanding of their condition was seen as enabler. This suggests that emphasizing this benefit may help increase trial participation and enhance participant satisfaction. As well, receiving the results of the study once they were complete was considered an important enabler towards participating in a trial. Providing findings to research participants will allow them to be informed about what was learnt from the trial and how their participation may have benefited the knowledge gained.^12^

### Limitations

Our study has several limitations that should be considered. The chief limitation stemmed from the very low response rate to the online survey component of our study (we received only 22 fully completed surveys from a network with over 2000 patients, family members and other interest holders, despite considerable effort), which limits our confidence that the survey results generalize to the full membership of SCAGO. Additionally, our respondents that completed the online survey were highly educated (93% with an undergraduate degree), thus not being fully representative of the broader Canadian sickle cell community. While our goal was to adhere to survey best practices,^30^ community practices and resources available to us required several deviations, such as indirect notification through social media and monthly newsletters, and few direct reminders. This work did not have the resources to offer incentives or send out personalized mailings to all members of the SCD communities. Additionally, competing surveys and priorities were being presented to the SCAGO community during the time our survey was taking place. While our approach engaged members of SCAGO throughout the development of the survey, and while the vast majority of those starting the survey completed it, both indicating a patient-relevant survey, it was clear that more resource and effort was needed to increase awareness among the broader population; future efforts could also broaden the sample frame to include other international SCD organizations.

Second, our survey questions were framed around a generic clinical trial scenario (“*imagine if you are asked today to participate in a clinical trial involving how well a new treatment works for SCD*”) rather than a specific SCD trial. A more specific recruitment scenario would likely elicit more specific barriers and enablers to trial participation and better capture participants’ intention to participant in a trial—a key predictor of actual participation.^17^ During our interview phase with members of SCAGO, we piloted the inclusion of a written description of an actual SCD trial; however, it became apparent that this would not be feasible for an online survey due to the added length of the survey and complexity. The inclusion of an actual trial description, perhaps as part of qualitative interviews rather than surveys, might suggest an alternative use of this theory-informed approach when specific trial characteristics are thought to be of particular importance for trialists.

Finally, the issue of mistrust of research has been reported to be a significant barrier within the SCD community,^2^^,^^10–12^^,^^14^ at least in part stemming from poor past treatment of the community in both research and medical care settings.^13^^,^^31–33^ Despite our efforts to engage the community during survey development and obtain the cooperation and endorsement of SCAGO, our low response rate may partly stem from a need for further relationship-building within the community. A relevant model might be that of the American Society of Hematology Research Collaborative, which created an initiative that involved the SCD community from the outset of the research enterprise, including identifying research priorities and ways to improve research participation.^14^

## CONCLUSIONS

We designed and implemented an approach to assessment of barriers to and enablers of trial participation in SCD that is informed by engagement with individuals who have lived experience of SCD, and explicitly guided by theory of human behavior change. This approach uncovers detailed insights into the challenges related to SCD trial participation, and make clear suggestions for addressing them based on existing behavior change literature. The approach is flexible, implementable via interviews or surveys, and can be conducted prior to trial onset to ensure optimized trial participation.

## Supplementary Material

yoaf032_Supplementary_Data

## Data Availability

The datasets used and/or analyzed during the current study are available from the corresponding author on reasonable request.
