# Supplementary material for: A patient-centered, theory-guided approach to examining the barriers and enablers to trial participation among people with SCD
Source: J Sick Cell Dis. 2025 Oct 7;2(1):yoaf032. doi: 10.1093/jscdis/yoaf032 (PMC12529103; doi:10.1093/jscdis/yoaf032)
Supplement: yoaf032_Supplementary_Data [file yoaf032_supplementary_data.docx]

**Supplementary Appendix Material. Table 1. Survey Development.**

**A Patient-Centered, Theory Guided Approach to Examining the Barriers and Enablers to Trial Participation amongst People with Sickle Cell Disease**

Kelly Carroll^1^, 0000-0002-7367-0270, MA, Natasha Hudek^1^, 0000-0002-3486-7871, PhD, Justin Presseau^1;2^, 0000-0002-2132-0703, PhD, Lanre Tunji-Ajayi^3^, 0009-0005-3375-1737, MSM, Dawn P. Richards^4^, 0000-0003-1151-0826, PhD, Susan Marlin^4^, 0000-0001-8971-7478, MSc, Jamie C. Brehaut^1;2*^, 0000-0002-4213-1143, PhD

^1^ Methodological and Implementation Research, Ottawa Hospital Research Institute (OHRI), The Ottawa Hospital, General Campus, 501 Smyth Rd, Ottawa, ON K1H 8L6, Canada; kecarroll@ohri.ca (K.C.); nhudek@ohri.ca (N.H), jbrehaut@ohri.ca (JCB), jpresseau@ohri.ca (J.P.)

^2^ School of Epidemiology and Public Health, University of Ottawa, 600 Peter Morand Crescent, Ottawa, ON K1G 5Z3, Canada

^3^ Sickle Cell Awareness Group of Ontario (SCAGO), 330-5109 Steeles Ave W, North York, ON M9L 2Y8, Canada; [sicklecellawarenessontario@gmail.com](mailto:sicklecellawarenessontario@gmail.com) (L.T.A)

^4^ Clinical Trials Ontario, 661 University Avenue, Suite 460, MaRS Centre, West Tower,

Toronto, ON M5G 1M1, Canada; dawn.richards@ctontario.ca (D.P.R.); susan.marlin@ctontario.ca (S.M.)

*Corresponding author: Jamie C. Brehaut ([jbrehaut@ohri.ca](mailto:jbrehaut@ohri.ca))

| **HD survey items (47 items)** | **First iteration Sickle Cell survey items (50 items)** | **Changes made during pilot** | **Final iteration Sickle Cell survey items (49 final items)** |
| --- | --- | --- | --- |
| 1. My belief that I’d learn more about my condition if I participated. | 1. My belief about whether I’d learn more about my condition if I participated | Modified for clarity | 1. My belief about whether I’d learn more about my condition if I participated |
| 1. If the trial were testing a very new untested treatment. | 1. If the trial were testing a very new untested treatment | No changes | 1. If the trial were testing a very new untested treatment |
| 1. If I find the trial documents hard to understand. | 1. If I find the trial documents hard to understand | No changes | 1. If I find the trial documents hard to understand |
| 1. If the consent documents describe probabilities of side effects and numbers of patients affected by them. | 1. If the consent documents describe probabilities of side effects and numbers of patients affected by them | No changes | 1. If the consent documents describe probabilities of side effects and numbers of patients affected by them |
| 1. My belief that participation is part of my role as a good citizen. | 1. My belief that participation is part of my role as a good citizen | Modified for clarity | 1. My belief that participation is part of being a good citizen |
| 1. My belief that participating would give me a sense of purpose. | 1. My belief that participating would give me a sense of purpose | No changes | 1. My belief that participating would give me a sense of purpose |
| 1. If I think it would be a challenge getting from my home to the study site. | 1. If I think it would be a challenge getting from my home to the study site | No changes | 1. If I think it would be a challenge getting from my home to the study site |
| 1. If I think my overall health is good. | 1. If I think my overall health is good. | No changes | 1. If I think my overall health is good. |
| 1. My belief that participating would give me a sense of control over what is happening to me. | 1. My belief that participating would give me a sense of control over what is happening to me | No changes | 1. My belief that participating would give me a sense of control over what is happening to me |
| 1. My feelings about the quality of my drug plan. | 1. My feelings about the quality of my drug plan | No changes | 1. My feelings about the quality of my drug plan |
| 1. My hope that participation would help find a cure. | 1. My hope that participation would help find a cure | No changes | 1. My hope that participation would help find a cure |
| 1. My belief that I would receive better care if I participated. | 1. My belief that I would receive better care if I participated | No changes | 1. My belief that I would receive better care if I participated |
| 1. My hope that participation will help me with my condition. | 1. My hope that participation will help me with my condition | No changes | 1. My hope that participation will help me with my condition |
| 1. My belief that participating would contribute to science. | 1. My belief that participating would contribute to science | No changes | 1. My belief that participating would contribute to science |
| 1. My belief that participating would help others. | 1. My belief that participating would help others | Modified for clarity | 1. My belief that participating in a trial would help others |
| 1. If I had to have more blood tests. | 1. If I had to have more tests | Modified for clarity/clinical relevance | 1. If I had to have more tests |
| 1. If I had to have more difficult tests (e.g., lumbar punctures, MRIs). | 1. If I had to have more invasive tests/procedures (e.g., bone marrow transplant) | Modified for clinical relevance | 1. If I had to have more invasive tests/procedures (e.g., bone marrow transplant) |
| 1. If I had to stay longer in hospital | 1. If I had to stay longer in hospital | No changes | 1. If I had to stay longer in hospital |
| 1. My worry that my insurance coverage would be affected. | 1. My worry that my insurance coverage would be affected | No changes | 1. My worry that my insurance coverage would be affected |
| 1. If participation meant that I would need to know my genetic status. |  | Deleted due to lack of clinical relevance |  |
| 1. My worry that participation will cause more work for my family. | 1. My worry that participation will cause more work for my family | No changes | 1. My worry that participation will cause more work for my family |
| 1. My experience with previous trials. | 1. My experience with previous trials | No changes | 1. My experience with previous trials |
| 1. If I would gain access to new study drugs. | 1. If I would gain access to new study drugs | No changes | 1. If I would gain access to new study drugs |
| 1. If the study reimbursed expenses. | 1. If the study reimbursed expenses | Modified to include an example | 1. If the study reimbursed expenses (e.g., parking) |
| 1. If I were paid for my participation. | 1. If I were paid for my participation | No changes | 1. If I were paid for my participation |
| 1. If I received the results of the study once it was complete. | 1. If I received the results of the study once it was complete | No changes | 1. If I received the results of the study once it was complete |
| 1. My belief that participation would prevent me from my other activities. | 1. My belief that participation would prevent me from my other activities | No changes | 1. My belief that participation would prevent me from my other activities |
| 1. My belief that participation would interfere with other goals of mine. | 1. My belief that participation would interfere with other goals of mine | No changes | 1. My belief that participation would interfere with other goals of mine |
| 1. If I think participation would affect my social life/family commitments. | 1. If I think participation would affect my social life/family commitments | No changes | 1. If I think participation would affect my social life/family commitments |
| 1. If I think participation would interfere with my child care responsibilities. | 1. If I think participation would interfere with my childcare responsibilities | No changes | 1. If I think participation would interfere with my childcare responsibilities |
| 1. If I think participation would affect my activities with other clinical trials. | 1. If I think participation would affect my activities with other clinical trials | No changes | 1. If I think participation would affect my activities with other clinical trials |
| 1. If the investigators provided telephone reminders about study appointments. | 1. If the investigators provided telephone reminders about study appointments | No changes | 1. If the investigators provided telephone reminders about study appointments |
| 1. My feelings about the quality of the health care system. | 1. My feelings about the quality of the health care system | No changes | 1. My feelings about the quality of the health care system |
| 1. If I think there is a substantial time commitment. | 1. If I think there is a substantial time commitment | No changes | 1. If I think there is a substantial time commitment |
| 1. If there was patient-friendly decision-making tools to help you make your participation decision. | 1. If there were patient-friendly decision-making tools to help you make your participation decision | No changes | 1. If there were patient-friendly decision-making tools to help you make a participation decision |
| 1. If the study provided transportation to/from study appointments. | 1. If the study provided transportation to/from study appointments | No changes | 1. If the study provided transportation to/from study appointments |
| 1. If my physician thought I should participate. | 1. If my physician(s) thought I should participate | No changes | 1. If my physician(s) thought I should participate |
| 1. If my family thought I should participate. | 1. If my family thought I should participate | No changes | 1. If my family thought I should participate |
| 1. My worry that participation would mean that family members would find out about my condition. 2. My worry that participation would mean that friends and/or co-workers would find out about my condition. | 1. My worry that participation would mean that family members would find out about my condition 2. My worry that participation would mean that friends and/or co-workers would find out about my condition. | Modified into a single item | 1. My worry that participation would mean that others would find out about my condition (e.g., friends, family, co-workers) |
| 1. My feelings about whether the trial funders can be trusted | 1. My feelings about whether the trial funders can be trusted | No changes | 1. My feelings about whether the trial funders can be trusted |
| 1. If there were helpful people on hand to help you make your participation decision. | 1. If there were helpful people on hand to help you make your participation decision | No changes | 1. If there were helpful people on hand to help you make a participation decision |
| 1. If the investigators provided regular study updates. | 1. If the investigators provided regular study updates about the trial | Modified for clarity | 1. If the investigators provided regular study updates about the trial |
| 1. If my physician was paid to recruit patients into the study. | 1. If my physician was given financial support to recruit patients into the study | Modified for clarity | 1. If my physician was given financial support to recruit patients into the study |
| 1. If there were a support person to help throughout the trial. | 1. If there were study staff to provide support throughout the trial | Modified for clarity | 1. If there were study staff to provide support throughout the trial |
| 1. My worry about unknown side effects. | 1. My worry about unknown side effects | No changes | 1. My worry about unknown side effects |
| 1. My belief that trial participation would help me plan how to manage treatment better. | 1. My belief that trial participation would help me plan how to manage treatment better | No changes | 1. My belief that trial participation would help me plan how to manage treatment better |
|  | 1. If Sickle Cell patient organizations endorse trial participation | New item added; modified | 1. If Sickle Cell patient organizations support trial participation |
|  | 1. If people I follow on social media support trial participation | New item added | 1. If people I follow on social media support trial participation |
|  | 1. My level of trust in the research team | Item added and then DELETED – not having an example to draw from makes it difficult to answer – really depends on who the research team is. |  |
|  | 1. If the study staff shares my cultural background | Item added and then DELETED – this was seen as a potentially distracting item. |  |
|  |  | New item added | 1. If the study documents mention a risk of death during the study |
|  |  | New item added | 1. If there was access to study staff that speaks my native language |
